# Arbeitsbedingte psychische Belastungen im Öffentlichen Gesundheitsdienst während und nach der COVID-19-Pandemie sowie Maßnahmen zur Reduktion

**DOI:** 10.1007/s00103-025-04164-9

**Published:** 2025-12-01

**Authors:** Kerstin Schmidt, Valentin Assheuer, Margarethe Kubitza, Isabell Schultheis, Susann Schmidt, Uwe Stengele, Dirk Cremer, Anke Ahäuser, Birte Pantenburg, Stella Duwendag, Kayvan Bozorgmehr, Brigitte Joggerst

**Affiliations:** 1 AG 2 Bevölkerungsmedizin und Versorgungsforschung, Universität Bielefeld, Universitätsstr. 25, 33615 Bielefeld, Deutschland; 2 Stadt Dortmund, Gesundheitsamt, Dortmund, Deutschland; 3 Kreis Herford, Gesundheitsamt, Herford, Deutschland; 4 Gesundheitsreferat (GSR), Landeshauptstadt München, München, Deutschland; 5 Landratsamt Pforzheim Enzkreis, Gesundheitsamt, Pforzheim, Deutschland; 6 Gesundheits‑, Veterinär- und Lebensmittelüberwachungsamt, Stadt Bielefeld, Bielefeld, Deutschland; 7 Institut für Sozialmedizin, Arbeitsmedizin und Public Health, Universitätsklinikum Leipzig, Leipzig, Deutschland; 8 Gesundheitsamt, Stadt Leipzig, Leipzig, Deutschland; 9 Public Health Nutrition, Universität Paderborn, Paderborn, Deutschland; 10 Gesundheitsamt, Landratsamt Karlsruhe, Karlsruhe, Deutschland

**Keywords:** Arbeitsplatzbezogene Belastung, ÖGD, Krisen, Partizipative Studie, Qualitative Interviews, Workplace-related stress, Public health service, Crises, Participatory study, Qualitative interviews

## Abstract

**Einleitung:**

Die neue Normalität nach der COVID-19-Pandemie ist für Mitarbeitende im Öffentlichen Gesundheitsdienst (ÖGD) von Unsicherheiten durch Herausforderungen wie geopolitische Bedrohungen, den Klimawandel und mögliche Pandemien sowie strukturelle Veränderungen durch die Digitalisierung geprägt. Die vorliegende qualitative Studie untersucht arbeitsplatzbezogene psychische Belastungen von Mitarbeitenden in Gesundheitsämtern während und nach der COVID-19-Pandemie sowie die Wirksamkeit von Maßnahmen, die diesen entgegenwirken sollen.

**Methoden:**

In einem partizipativen Verfahren zwischen der Universität Bielefeld und 7 Gesundheitsämtern wurden 15 im Herbst und Winter 2023/2024 geführte qualitative Interviews inhaltsanalytisch nach Mayring ausgewertet. Die Mitarbeiter:innen in den Gesundheitsämtern wurden nach Belastungsfaktoren in ihrem Arbeitsalltag während und nach der COVID-19-Pandemie gefragt und danach gebeten, von den Arbeitgebern durchgeführte Maßnahmen zur Reduktion dieser Belastungen zu benennen und ihre Wirksamkeit einzuschätzen.

**Ergebnisse:**

Als Hauptbelastungen wurden ein überhöhter Arbeitsumfang und fachfremde Aufgaben, die im Kontext der Pandemie entstanden, identifiziert. Die von den Arbeitgebenden ergriffenen Maßnahmen waren meist nicht auf die Belastungen abgestimmt und reduzierten diese nicht nachhaltig. Viele Befragte sahen sich daher nicht in der Lage, bei künftigen Krisen adäquat zu handeln. Als authentisch empfundene Wertschätzung wurde jedoch durchgehend positiv bewertet und als entlastend wahrgenommen.

**Diskussion:**

Die Ergebnisse zeigen angesichts der Möglichkeit künftiger Krisen und Veränderungen die dringende Notwendigkeit einer verbesserten Vorbereitung. Diese sollte insbesondere die Bedürfnisse der Beschäftigten im ÖGD mit einbeziehen.

## Abstract (en)

**Introduction:**

The new normal following the COVID-19 pandemic is characterized by uncertainty for employees in the public health service (ÖGD), shaped by challenges such as geopolitical threats, climate change, potential future pandemics, and structural changes driven by digitalization. The present qualitative study examines work-related psychological stress factors among public health office employees during and after the COVID-19 pandemic as well as the effectiveness of measures implemented to counter these stressors.

**Methods:**

In a participatory process between Bielefeld University and seven public health offices, 15 qualitative interviews conducted in autumn and winter 2023/2024 were analyzed using Mayring’s qualitative content analysis. The public health employees were asked about stress factors in their daily work during and after the COVID-19 pandemic and were then invited to identify employer-initiated measures aimed at reducing these burdens and to assess their effectiveness.

**Results:**

The main stressors identified were excessive workload and tasks outside the employees’ professional expertise that had emerged in the context of the pandemic. The measures implemented by employers were generally not well aligned with these burdens and therefore did not reduce them sustainably. Many respondents felt insufficiently prepared to act appropriately in future crises. However, expressions of authentic appreciation were consistently rated positively and perceived as relieving.

**Discussion:**

Given the likelihood of future crises and ongoing transformations, the results highlight an urgent need for improved preparedness. Such efforts should place particular emphasis on addressing the needs and perspectives of employees within the ÖGD.

## Einleitung

Die zentrale Rolle des Öffentlichen Gesundheitsdienstes (ÖGD) beim Schutz der Gesundheit der Bevölkerung wurde insbesondere während der COVID-19-Pandemie deutlich [1]. Gleichzeitig wurden während der Pandemie bekannte strukturelle Schwächen erneut sichtbar, etwa in Bezug auf eine angespannte Personaldecke, unzureichende Stellenplanung und nicht konkurrenzfähige Gehälter, die die Rekrutierung von Fachkräften erschwerten [2], sowie die mangelhafte Digitalisierung. Zusammen mit der enormen Belastung durch die Pandemie selbst führten die strukturellen Schwächen für Teile der Belegschaft zu Überlastung bis hin zu Erkrankungen [3].

Nach der Bewältigung der pandemiebedingten Herausforderungen sehen sich die Mitarbeitenden des ÖGD mit zusätzlichen Aufgaben konfrontiert. Dazu zählen die Vorbereitung auf künftige Pandemien, die zunehmende Digitalisierung, der demografische Wandel mit veränderten Anforderungen an die Gesundheitsversorgung sowie die oft unvorhersehbaren Auswirkungen des Klimawandels und geopolitische Bedrohungen. Zwar hat der im September 2020 von Bund und Ländern zur Verbesserung der personellen und strukturellen Kapazitäten im ÖGD beschlossene „Pakt für den ÖGD“ Reformprozesse zur Verbesserung bestehender Strukturen angestoßen, deren nachhaltige Auswirkungen müssen sich jedoch noch zeigen [4]. In diesem Spannungsfeld aus externen und internen Belastungsfaktoren entwickelt sich für die Mitarbeitenden des ÖGD eine neue, von Unsicherheiten geprägte Normalität nach der COVID-19-Pandemie.

Empirische Befunde zu arbeitsplatzbezogenen Belastungen und Gegenmaßnahmen im ÖGD sind bislang kaum vorhanden. Die vorliegende qualitative Studie hat explorativen Charakter und erlaubt Einblicke in die Wahrnehmungen einiger Mitarbeitender im ÖGD, die jedoch nicht notwendigerweise auf die Gesamtpopulation der ÖGD-Beschäftigten übertragbar sind. Ziel war es daher, vertiefende Erkenntnisse über arbeitsplatzbezogene Belastungserfahrungen von Mitarbeitenden im ÖGD zu gewinnen.

Es wurden folgende Forschungsfragen untersucht: 1) Wie erlebten die Mitarbeitenden die Belastungen während der COVID-19-Pandemie und wie schätzen sie diese im Vergleich zur Situation vor der Pandemie und zum Zeitpunkt des Interviews nach der Pandemie ein? 2) Wie bewerten die Mitarbeitenden die Maßnahmen ihrer Arbeitgebenden (Gesundheitsämter, Landratsämter oder Stadtverwaltungen), die während der Pandemie ergriffen wurden, um die Gesundheit der Beschäftigten zu erhalten, ihre Arbeit wertzuschätzen und der psychischen Belastung angemessen zu begegnen?

Im Folgenden wird zunächst das Studiendesign erläutert. Danach werden die von den Befragten genannten Belastungsfaktoren und Maßnahmen vorgestellt. Aufbauend auf den Bewertungen der ergriffenen Maßnahmen ihrer Arbeitgebenden werden Handlungsempfehlungen zur Reduktion dieser Belastungen abgeleitet, deren Wirksamkeit in anderen Kontexten zu eruieren ist.

## Methoden

Im Rahmen des Forschungs‑, Trainings- und Evidenznetzwerks für die Öffentliche Gesundheit – ÖGD-FORTE [5, 6] wurde das Thema „arbeitsplatzbezogene psychische Belastung von Mitarbeitenden im ÖGD“ als praxisrelevantes Forschungsthema identifiziert. Das daraus hervorgegangene Forschungsdesign der „Psych-ÖGD-Studie“ (Arbeitsplatzbezogene psychische Belastung von Mitarbeiter:innen des Öffentlichen. Gesundheitsdiensts im Zeitverlauf: eine mixed-methods Studie) [7] wurde in Zusammenarbeit zwischen der Universität Bielefeld und mehreren Gesundheitsämtern weiterentwickelt und durchgeführt. Die Studie folgt einem vertiefenden sequenziellen Mixed-Methods-Ansatz in der Form einer Längsschnittstudie, die bisher 2 quantitative und eine qualitative Datenerhebung umfasst.

Die qualitativen Ergebnisse der Studie, die im Folgenden vorgestellt werden, basieren auf 15 Interviews, die einmalig im Herbst und Winter 2023/2024 durchgeführt wurden. Dabei wurden Vertreter:innen verschiedener Berufsgruppen, Altersgruppen und Erfahrungsstufen aus 7 Gesundheitsämtern in ländlichen und städtischen Regionen Deutschlands anhand von semistrukturierten Leitfadeninterviews befragt. Einschlusskriterien waren die Volljährigkeit sowie eine zeitlich befristete oder unbefristete Beschäftigung bei den teilnehmenden Gesundheitsämtern zum Zeitpunkt des Interviews. Der Aufruf zur Studienteilnahme wurde an den teilnehmenden Gesundheitsämtern durch die dortigen Projektkoordinator:innen per E‑Mail an die gesamte Belegschaft versendet. Aus Datenschutzgründen erfolgte die Rückmeldung direkt an das Team der Universität Bielefeld, sodass die Namen der Studienteilnehmer:innen einzig der universitären Projektkoordination bekannt sind. Die Auswahl der Teilnehmenden aus 31 eingegangenen Interessensbekundungen wurde so getroffen, dass aus jedem teilnehmenden Amt maximal 3 Personen interviewt wurden.

Im Vorfeld der Interviews wurde ein Leitfaden mit 4 Themenkomplexen entwickelt, an denen sich die Interviews orientierten: 1. Faktoren, die zu arbeitsplatzbezogener psychischer Belastung führen; 2. Auswirkungen der Belastungen (beruflich und privat); 3. individuelle Strategien zur Reduktion der Belastung; 4. strukturelle Maßnahmen zur Reduktion der Belastung.

Die Interviews wurden nach Einholung einer schriftlichen Einwilligung von Forschenden der Universität Bielefeld via Zoom durchgeführt und als Audio-Aufzeichnung lokal auf deren Arbeitsgeräten gespeichert. Die 30- bis 90-minütigen Audio-Dateien wurden mithilfe eines inhaltlich-semantischen Transkriptionssystems in Anlehnung an das inhaltlich-semantische Transkriptionsmodell nach Dresing und Pehl [8] als Volltexte transkribiert und nach der Methode der qualitativen Inhaltsanalyse [9] ausgewertet.

Die Kodierung erfolgte mit der Software QCAmap (Verein zur Förderung qualitativer Forschung, Klagenfurt, Österreich) in einem partizipativen Verfahren mit Forschenden der Universität Bielefeld und 7 an der Entwicklung und Durchführung der Psych-ÖGD-Studie teilnehmenden Gesundheitsämtern. In Kleingruppen wurden relevante, anonymisierte Interviewausschnitte diskutiert, interpretiert und anhand der Forschungsfragen kodiert. Danach wurden die gewählten Codes und ihre Bedeutung in der Gesamtgruppe der Teilnehmenden besprochen und ein einheitliches Kodiersystem mittels induktiver Kategorienbildung erarbeitet. Dies ermöglichte die Reduzierung des Materials auf die für die Studie relevanten Inhalte. Nach der Durcharbeitung von 50 % des Materials wurde eine gemeinsame Rücküberprüfung des Kodiersystems durchgeführt. Abschließend wurden die Interviewausschnitte mithilfe des finalen Kodiersystems erneut bearbeitet (Tab. 1 für die finalen Kategorien der Belastungsfaktoren und Maßnahmen). Somit konnte eine möglichst hohe Intercoder-Reliabilität erreicht werden. Die Darstellung der Ergebnisse orientiert sich an den konsolidierten Empfehlungen zur Berichterstattung qualitativer Forschung (COREQ; [10]).

**Tab. 1:** Kategorien der Belastungsfaktoren und Maßnahmen

Belastung Kernthemen	Belastung durch den Arbeitsumfang
	Belastung durch den Arbeitsinhalt
Weitere Belastungsfaktoren	Missachtung des Arbeitsschutzes
	Anspruchshaltung an die eigene Arbeit
	Mangelnde Selbstwirksamkeit
	Konflikt zwischen Pandemie- und Fachaufgaben
	Aufgabenspektrum
	Geringe Wertschätzung durch die Bürger
	Digitalisierung als zusätzliche Belastung
	Ungleich verteilte Arbeitsbelastung
	Einsatzbereitschaft für künftige Pandemien
	Fehlende Kollegialität
	Gesundheitliche Auswirkungen
	Vorbereitung auf eine kommende Pandemie
	Bezahlung im ÖGD
	Fehlende Ansprechpersonen/intransparente Führungsstrukturen
	Wunsch nach individuellem Austausch mit Vorgesetzten
	Kommunikation der strategischen Ausrichtung des GA
	Unzufriedenheit mit der Arbeitsplatzgestaltung
	Wunsch nach strukturellem Austausch
	Stellenplanung und Mitarbeitermangel
	Starre und langsame hierarchische Strukturen
	Fehlende Transparenz der Arbeitsanweisungen
	Mangelnde Digitalisierung
Maßnahmen	Maßnahme: flexibles Arbeiten
	Maßnahme: interner Austausch und Reflexion
	Maßnahme: Mitarbeiterevent
	Maßnahme: Geschenk
	Maßnahme: schriftliche Anerkennung
	Maßnahme: Gesundheitskurse
	Maßnahme: Beratungsstelle
	Maßnahme: Bewirtung
	Maßnahme: pauschale Corona-Prämien und Überstundenzuschläge

## Ergebnisse

Es wurden 11 weibliche und 4 männliche Mitarbeitende aus Gesundheitsämtern an den Standorten Bielefeld, Dortmund, Herford, Leipzig, München, Pforzheim und Potsdam befragt. Unter den Teilnehmenden befinden sich Personen mit und ohne Leitungsaufgaben in unterschiedlichen Berufsgruppen.

### Belastungsfaktoren

Ein hoher Arbeitsumfang und ein belastender Arbeitsinhalt wurden von der großen Mehrheit der Befragten als Hauptursachen für die arbeitsplatzbezogene psychische Belastung während der Pandemie identifiziert. Im Folgenden werden daher die in diesen beiden Oberkategorien zusammengefassten Ergebnisse dargestellt und durch Zitate illustriert.

#### Belastung durch hohen Arbeitsumfang

Der Arbeitsumfang, der allgemein als zu hoch und oft auch als ungleich verteilt wahrgenommen wurde, stellte für die Mehrheit der Befragten eine wichtige Belastungsursache dar. In vielen Fällen führte dies dazu, dass sie trotz großer Leistungsbereitschaft und Freude an der Tätigkeit ihrem eigenen Anspruch an die Arbeit im Rahmen der regulären Arbeitszeit nicht mehr gerecht werden konnten, wie das folgende Zitat illustriert:

„Es macht ja auch Spaß, aber es ist zu viel Arbeit, … ich kriege das nicht in einer normalen Arbeitswoche hin, also, zumindest nicht mit dem Anspruch, dass es gut ist und insofern … ist es gerade sehr, sehr stressig …“ (Interview 6).

Durch die Pandemie wurde dieser Eindruck verstärkt, wie das folgende Zitat zeigt:

„Das hat bis zu einer Sieben-Tage-Woche geführt“ (Interview 2).

Dies führte bei einigen Befragten zu gesundheitlichen Auswirkungen. Mechanismen des Arbeitsschutzes, aber auch Fürsorgemaßnahmen für die Mitarbeitenden griffen in der Pandemie nicht mehr. Das folgende Zitat verdeutlicht dies:

„Also so die ersten anderthalb Jahre habe ich am Ende mit so einer [chronischen Erkrankung] bezahlt“ (Interview 3).

Einige der Mitarbeitenden äußerten eine fehlende Bereitschaft, sich im Falle einer weiteren Pandemie erneut in vergleichbarem Maße zu engagieren, oder warnten vor den Folgen langfristiger Überbelastung der Mitarbeitenden im ÖGD.

„… ich habe mein Soll erfüllt von 2020 bis 2022“ (Interview 6).

„… wenn die Leute alle rund geschliffen sind, ganz deutlich gesagt, wird das eng, weil so viel Nachwuchs gibt es nicht“ (Interview 5).

#### Belastung durch den Arbeitsinhalt

Das zweite zentrale Thema ist eine hohe Belastung durch den Arbeitsinhalt. Dieser Bereich ist stark verknüpft mit den Herausforderungen der Pandemie. Viele Befragte berichteten von ihrer Verantwortung für fachfremde Aufgaben, die im Kontext der Pandemie entstanden und die als besonders belastend wahrgenommen wurden. Oftmals bemängelten die Mitarbeitenden in diesem Kontext eine fehlende Selbstwirksamkeit bei ihrer Tätigkeit.

„… dass man eben manche Dinge jetzt vorrangig behandeln muss und andere Dinge, wo man vorher … so gut daran gearbeitet hat, dann eben runterfallen lassen musste“ (Interview 14).

„Mitarbeiter oder Mitarbeiterinnen … die das total stressig fanden, die auch Angst hatten, die Leute falsch zu beraten, weil die sich plötzlich mit so einer Aufgabe, die sie noch nie gemacht hatten, überfordert fühlten“ (Interview 10).

Auch die zur Erleichterung der Arbeit in einigen Ämtern vorangetriebene Digitalisierung wurde von Teilen der Belegschaft durch die damit einhergehenden neuen Herausforderungen und Unsicherheiten in Bezug auf zukünftige Entwicklungen als belastend empfunden, wie dieses Zitat verdeutlicht:

„Ich liebe meinen Job. Aber durch diese Digitalisierung und diesen Wandel, sehne ich mich nach Rente wie nie zuvor“ (Interview 9).

In verschiedenen Interviews offenbarte sich, dass eine während der Pandemie als unzufriedenstellend empfundene Kommunikation einen Belastungsfaktor darstellt. Dazu gehört der Wunsch nach mehr Austausch sowohl im Team als auch im direkten Kontakt zu den Vorgesetzten. Weitere kritisierte Aspekte waren eine intransparente Kommunikation von Arbeitsanweisungen sowie von Zielen und der langfristigen Strategie des Amtes, was zu einem Gefühl des „Nicht-Eingebundenseins“ führte und mit mangelnder Wertschätzung in Verbindung gebracht wurde.

„Und da vielleicht einfach mal auf einer halben Seite zusammenzufassen: Das ist im Moment in unseren Überlegungen und so wollen wir weiterverfahren. Das hätte ich als wertschätzend empfunden, nämlich, dass man sozusagen wichtig genug ist, informiert zu werden“ (Interview 3).

Diese Wahrnehmung trat in erster Linie bei Personen ohne Leitungsfunktion auf. Mitarbeitende, die selbst eine Leitungsfunktion innehatten, berichteten ihrerseits davon, dass die stark hierarchisch geprägten Führungsstrukturen im ÖGD sie immer wieder in ihren Handlungsspielräumen begrenzten.

„Das fällt mir hier manchmal etwas schwer, dass die Strukturen und die Mühlen sehr langsam mahlen und wirklich auch immer nur in eine Richtung“ (Interview 12).

Mehrere Befragte äußerten infolge ihrer Erfahrungen während der Pandemie verschiedene Vorstellungen, durch welche strukturellen Veränderungen bei der Vorbereitung auf eine kommende potenzielle Krise die Belastung der Mitarbeitenden reduziert werden könnte. Eine zentrale Forderung war hierbei die stärkere Fokussierung auf zukünftige Herausforderungen.

„… dass man nicht immer reagiert auf Dinge … Sondern dass man sich vielleicht manchmal ein bisschen aufs Agieren und aufs mehr vorausschauende Handeln verlegen würde“ (Interview 12).

In der Rückschau auf die Zeit während der Pandemie waren die Antworten der Befragten häufig von starken Emotionen geprägt, die sich besonders im Hinblick auf die eigenen Belastungsgrenzen zeigten. Ein zentrales Ergebnis der Studie ist daher, dass die psychische Belastung primär durch den Arbeitsumfang und den Arbeitsinhalt bestimmt wird. Strukturelle, organisatorische und kommunikative Defizite im Arbeitskontext verstärkten die wahrgenommene Belastung in einigen Ämtern und Arbeitsbereichen.

### Bewertung von Maßnahmen

Von den Befragten wurden verschiedene Maßnahmen genannt, die sie selber zur Reduktion der arbeitsplatzbezogenen Belastung einsetzten. In der folgenden Darstellung liegt der Fokus jedoch auf Maßnahmen, die von den Arbeitgebenden oder übergeordneten Behörden eingesetzt wurden.

Am häufigsten wurden Maßnahmen genannt, die als Gesten der Anerkennung für die geleistete Arbeit generell oder für einen besonderen Arbeitseinsatz während der Pandemie verstanden werden können. Es zeigte sich, dass die Motivation für solche Maßnahmen, wie z. B. kleine Geschenke oder eine schriftliche Anerkennung, auch von Mitarbeitenden des gleichen Amtes sehr unterschiedlich eingeordnet wurde. Mitarbeitende, die keine persönliche Beziehung zu ihren Führungskräften hatten, konnten diesen Gesten eher wenig abgewinnen. So bewertete ein Befragter Geschenke an die gesamte Belegschaft und sagte im Hinblick auf die Bedeutung als Geste der Wertschätzung:

„Ich habe das in der Realität nie wahrgenommen“ (Interview 3).

Mitarbeitende, die den Umgang mit ihren Vorgesetzten jedoch generell eher positiv bewerteten, nahmen auch die Bedeutung von Gesten der Wertschätzung eher positiv auf.

„Wenn man diejenigen kennt, die das Schreiben geschrieben haben, dann weiß man auch, dass es so gemeint ist“ (Interview 4).

Durch diese Zitate zeigt sich, dass an die gesamte Belegschaft gerichtete Maßnahmen der Wertschätzung in ihrer Rezeption davon abhängig waren, ob die Mitarbeitenden sich auch im normalen Arbeitsalltag wertgeschätzt fühlen. Eine Ausnahme bildeten hierbei Mitarbeiterveranstaltungen, die zum internen Austausch anregten; diese Maßnahme wurde generell positiv aufgenommen. Eine nachhaltige Verbesserung der Belastungssituation konnte allerdings durch keine Maßnahme der Wertschätzung erreicht werden, was durch die folgende Einschätzung einer mit Leitungsaufgaben betrauten Person illustriert wird:

„… nur einmal einen großen Karton Pizza zu bestellen, dadurch sind die Leute nicht glücklich“ (Interview 11).

Maßnahmen zur Verhaltensprävention, wie z. B. spezifische Gesundheitsangebote, die der Stressreduktion dienen sollen und die von einigen Arbeitgebern im ÖGD angeboten werden, wurden in den Interviews selten genannt. Häufig wussten die befragten Mitarbeitenden auf Nachfrage nicht, ob es solche Angebote gibt und wie sie genutzt werden können, wie dieses Zitat illustriert:

„… so gesundheitsfördernde Angebote … habe ich nie so auf dem Schirm gehabt“ (Interview 13).

In den Fällen, in denen Angebote der betrieblichen Gesundheitsförderung bekannt waren, wurde das Kursangebot positiv bewertet, dennoch aber aus Zeitgründen oft nicht genutzt. Für die Befragten war es – insbesondere während der extremen Zeiten der Pandemie – entweder wichtiger, Zeit für die Familie und persönliche Anliegen zu haben, oder die Befragten waren stark auf die beruflichen Herausforderungen fokussiert.

Maßnahmen zur Verhältnisprävention, insbesondere zur Verbesserung der strukturellen Bedingungen, wurden von den Befragten, in den seltenen Fällen, in denen sie angesprochen wurden, eng mit der durch den Pakt für den ÖGD verbesserten Digitalisierung verknüpft. Positiv bewertet wurden Digitalisierungsmaßnahmen im Hinblick auf effizientere Arbeitsabläufe und Kommunikation sowie erweiterte Homeoffice-Möglichkeiten.

„Also die Digitalisierung ist im vollen Gange. … Da hatten wir jetzt Schulungen. Auch da werden wir vorbereitet und begleitet. Ich denke, da sind wir wirklich auf einem guten Weg“ (Interview 12).

Dennoch wurde der Stand der Digitalisierung weiterhin als defizitär bewertet, wie dieses Zitat verdeutlicht.

„Es arbeiten immer noch Gesundheitsämter mit Faxen. Und so sind wir ja auch noch damals in der Pandemie gestartet. Also das sind alles so Dinge, da hinken wir eben halt“ (Interview 8).

Die Ergebnisse zeigen, dass die von den Befragten beschriebenen Maßnahmen zwar primär auf die Entlastung der Mitarbeitenden abzielten, dabei jedoch häufig die den Oberkategorien des Arbeitsumfangs und -inhalts zugrunde liegenden Belastungsfaktoren nicht ausreichend adressierten. Eine nachhaltige Reduktion der arbeitsplatzbezogenen psychischen Belastung blieb somit aus.

## Diskussion

Die Ergebnisse zeigen, dass die Pandemie nachhaltige emotionale Spuren bei vielen Beschäftigten im ÖGD hinterlassen hat und dass konventionelle Maßnahmen zur Reduktion der psychischen Belastung, wie Belohnungen oder Entspannungsangebote, zu kurz greifen, um die teilweise massiven Belastungen durch die Arbeit in der Pandemie aufzufangen. Die Ergebnisse der Belastungserfahrung decken sich in wesentlichen Punkten mit bisherigen Studien zur arbeitsplatzbezogenen Belastung im Gesundheitswesen während der COVID-19-Pandemie, etwa der VOICE-Studie [4], in der Überlastung, Kontrollverlust und mangelnde Unterstützung als zentrale Stressoren identifiziert wurden. Gleichzeitig zeigt sich in der vorliegenden Untersuchung, dass die Belastungssituation im ÖGD durch spezifische strukturelle Probleme wie eine unzureichende Personalplanung, Kommunikations- und Digitalisierungsdefizite sowie eine geringe Selbstwirksamkeitserfahrung geprägt war. Diese bestanden oftmals bereits vor der Pandemie und verstärkten sich in der Krisenzeit durch eine erhöhte Arbeitsbelastung und den von einigen Befragten als anspruchsvoller wahrgenommenen Arbeitsinhalt.

Daraus folgt eine mangelnde Bereitschaft in Teilen der Belegschaft, in zukünftigen Krisensituationen zusätzliche Aufgaben zu übernehmen. Dies stellt in der aktuellen von Unsicherheiten u. a. im Hinblick auf die Auswirkungen des Klimawandels, geopolitische Bedrohungen sowie erneute Pandemien geprägten Realität eine große Herausforderung dar. Diese Herausforderung geht über die im „Pakt für den ÖGD“ identifizierten Maßnahmen hinaus, die eine Stärkung der Personalstruktur und attraktivere Gehaltsstrukturen anstreben [1, 4].

Die angebotenen Maßnahmen zur Reduktion der Belastung sind nicht an die Belastungen angepasst, sodass Maßnahmen zur Verhaltensprävention, wie Gesundheitsangebote, nur selten von den Beschäftigten angenommen wurden. Insgesamt zeigt sich, dass Maßnahmen zur Reduktion psychischer Belastung im ÖGD nicht nur kurzfristig ansetzen dürfen, sondern strukturelle Rahmenbedingungen berücksichtigen müssen, um wirksam zu sein. Die Frage, wie Mitarbeitende auf zukünftige Krisen vorbereitet werden können, erscheint daher als Ergebnis der Studie zentral.

Aufgrund des qualitativen Designs sind hier keine generalisierbaren Aussagen möglich, aber auch nicht beabsichtigt. Es gilt vielmehr, die Wahrnehmung Betroffener aussagekräftig empirisch zu reproduzieren und dabei möglichst vielfältige, kontextabhängige Perspektiven zu untersuchen. Darüber hinaus sind potenzielle Verzerrungen bei der retrospektiven Einschätzung arbeitsbezogener Belastungen vor, während und nach der COVID-19-Pandemie nicht auszuschließen.

## Fazit – Handlungsempfehlungen für die Praxis

**Fachkräftesicherung. **Zur langfristigen Sicherung der Fachkräfte im Öffentlichen Gesundheitsdienst erscheint eine weitere Stärkung der Personalstrukturen unerlässlich. Dies erfordert zum einen eine vorausschauende und bedarfsgerechte Stellenplanung, zum anderen attraktivere Gehaltsstrukturen, um qualifiziertes Personal verschiedener Berufsgruppen zu gewinnen und zu halten.

**Wertschätzung als Haltung und Kommunikation. **Die Ergebnisse verdeutlichen erneut, dass Wertschätzung primär als Haltung und nicht als isolierte Maßnahme zu verstehen ist. Diese Erkenntnis ist zwar Bestandteil einschlägiger Führungskräfteschulungen, erfordert jedoch eine kontinuierliche Reflexion und aktive Umsetzung. Eine gelebte Haltung der Wertschätzung trägt wesentlich zur langfristigen Förderung von Motivation und psychischer Gesundheit der Mitarbeitenden bei. Die Beobachtung, dass Interventionen insbesondere dann positiv rezipiert wurden, wenn bereits vor der Krise ein vertrauensvolles Verhältnis zur Führungskraft bestand, unterstreicht die Relevanz einer konsistent wertschätzenden Kommunikationskultur. Sie bildet die Grundlage dafür, dass Anerkennung als authentisch und glaubwürdig wahrgenommen wird.

**Krisenbewältigung. **Die Interviews zeigen, dass ein großer Anteil der Befragten auch nach Ende der Pandemie weiterhin emotional stark von der Krise betroffen ist. Zur Bewältigung dieser Belastungen erscheinen Austauschformate, ggf. auch mit professioneller Unterstützung, sinnvoll, deren Ausgestaltung mit den Beteiligten abgestimmt werden sollte. Auch die Akzeptanz des Nichtwissens und Nichtschaffens durch Mitarbeitende und Vorgesetzte kann zu einem besseren Umgang mit arbeitsplatzbezogener Belastung während Krisen beitragen.

**Digitalisierung. **Die fortschreitende Digitalisierung bietet Chancen, wird jedoch von vielen der Befragten bisher als ambivalent wahrgenommen. Schulungsangebote und fachgerechte Unterstützung bei der Implementierung können zusätzliche Belastungen verringern und die Entlastung durch die Verbesserung von Prozessabläufen fördern. Optimal ist eine Beteiligung der Mitarbeitenden bei der Entwicklung und Implementierung neuer Fachanwendungen.

## Data Availability

Die während der vorliegenden Studie erzeugten und analysierten Daten (qualitative Interviews) sind aus Datenschutzgründen nicht öffentlich zugänglich, können aber auf begründete Anfrage bei der Korrespondenzautorin angefordert werden.
